# *rac*-2-[2-(4-Fluoro­phen­yl)-2-oxo-1-phenyl­eth­yl]-4-methyl-3-oxo-*N*-phenyl­penta­namide

**DOI:** 10.1107/S1600536810046271

**Published:** 2010-11-13

**Authors:** Feng-yan Zhou, Jian-ying Huang

**Affiliations:** a Department of Chemistry, Zaozhuang University, Shandong, People’s Republic of China; b Department of Applied Chemistry, Zhejiang Gongshang University, Hangzhou 310035, People’s Republic of China

## Abstract

The title compound, C_26_H_24_FNO_3_, is a critical inter­mediate of a selective and competitive inhibitor of the enzyme 3-hy­droxy-3-methyl­glutaryl-coenzyme A (HMG–CoA) reductase. Inter­molecular N—H⋯O hydrogen bonding generates a chain along **[give direction]** that is the dominant inter­action in the crystal packing. Inter­molecular C—H⋯O inter­actions are also observed.

## Related literature

For related structures, see: Baumann *et al.* (1992 ▶). For the title compound as an inter­mediate in the preparation of the HMG-CoA reductase inhibitor atorvastatin, see: Roth *et al.* (1991 ▶); Wang *et al.* (2007 ▶). 
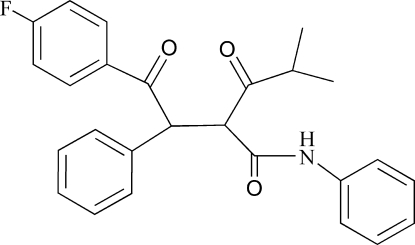


## Experimental

### 

#### Crystal data

C_26_H_24_FNO_3_
*M* *_r_* = 417.46
Monoclinic, 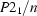

*a* = 14.1694 (14) Å
*b* = 9.8307 (9) Å
*c* = 16.6367 (16) Å
β = 99.651 (2)°
*V* = 2284.6 (4) Å^3^
*Z* = 4
Mo *K*α radiation
μ = 0.08 mm^−1^
*T* = 173 K
0.15 × 0.10 × 0.05 mm

#### Data collection

Bruker SMART CCD area-detector diffractometer
Absorption correction: multi-scan (*SADABS*; Bruker, 2005 ▶) *T*_min_ = 0.990, *T*_max_ = 0.996
10555 measured reflections
3792 independent reflections
2493 reflections with *I* > 2σ(*I*)
*R*_int_ = 0.027

#### Refinement

*R*[*F* ^2^ > 2σ(*F* ^2^)] = 0.067
*wR*(*F* ^2^) = 0.227
*S* = 1.06
3792 reflections
275 parameters
1 restraint
H-atom parameters constrained
Δρ_max_ = 0.78 e Å^−3^
Δρ_min_ = −0.68 e Å^−3^

### 

Data collection: *SMART* (Bruker, 2005 ▶); cell refinement: *SAINT* (Bruker, 2005 ▶); data reduction: *SAINT*; program(s) used to solve structure: *SHELXS97* (Sheldrick, 2008 ▶); program(s) used to refine structure: *SHELXL97* (Sheldrick, 2008 ▶); molecular graphics: *SHELXTL* (Sheldrick, 2008 ▶); software used to prepare material for publication: *SHELXL97*.

## Supplementary Material

Crystal structure: contains datablocks I, global. DOI: 10.1107/S1600536810046271/kp2271sup1.cif

Structure factors: contains datablocks I. DOI: 10.1107/S1600536810046271/kp2271Isup2.hkl

Additional supplementary materials:  crystallographic information; 3D view; checkCIF report

## Figures and Tables

**Table 1:** Hydrogen-bond geometry (Å, °)

*D*—H⋯*A*	*D*—H	H⋯*A*	*D*⋯*A*	*D*—H⋯*A*
N1—H1⋯O1^i^	0.88	2.10	2.959 (2)	178 (4)
C1—H1*A*⋯O3^ii^	0.95	2.43	3.371 (5)	169

Symmetry codes: (i) 
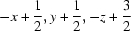
; (ii) 
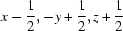
.

**Fig. 1.**
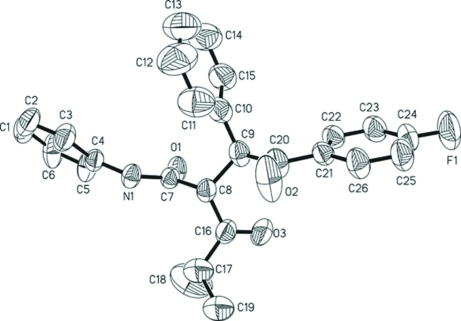
The molecular structure with atom labels and 45% probability displacement ellipsoids for non-H atoms.

**Fig. 2.**
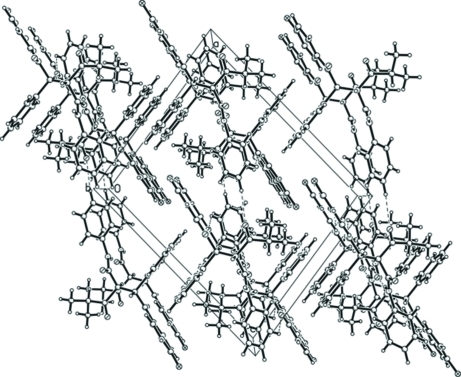
The packing diagram of molecular, viewed down the b axis, with the N—H···O interactions shown as dashed lines.

## supplementary crystallographic information

### Comment

The title compound, C26H24FNO3(I),(Fig. 1) is of value as an pharmaceutical intermediate, particularly an intermediate of an HMG-CoA reductase inhibitor, atorvastatin (Roth *et al.*, 1991; Wang *et al.*, 2007). Though this compound reveals an opposite chirality at C8 and C9, up to now, the absolute configuration has not been reported so far, enantiomers separation or sterespecific synthesis of enantiomer of the title compound. A racemic mixture is always directly used to prepare HMG-CoA reductase inhibitor. Using *N*-ethyl thiazolium bromide as the catalyst, reacting 4-fluorobenzaldehyde with benzylidine isobutyrylacetamide affords I, as a white solid (Baumann *et al.*, 1992). Suitable crystals of I for single-crystal X-ray analysis were obtained by a vapor diffusion method. In the crystal structure intermolecular hydrogen bond N –H···O is a dominant interaction forming a chain (Table 1).

### Experimental

2-Benzylidine isobutyrylacetamide (2.93 g, 10.0 mmol), *N*-ethyl thiazolium bromide (0.290 g, 1.50 mmol), 4-fluorobenzaldehyde (1.36 g, 11.0 mmol), and triethylamine (8.08 g, 8.00 mmol) were mixed and heated to 338 K. The reaction mixture was stirred for 24 h maintaining the temperature at 338 K. After 2-propanol (5 ml) and water (5 ml) were added, a white precipitate was formed. The precipitate was filtered, washed with 2-propanol, and dried to afford the title compound (2.09 g, 50.2%) as a white solid. Colourless crystals were obtained by vapor diffusion of pentane into an acetone solution over a period of 5 d. 1H NMR (500 MHz, CD3CN, 22 °C): δ 8.48 (1 H, s), 8.10 (2 H, m), 7.37 (2 H, m), 7.25 (6 H, m), 7.18 (3 H, m), 7.07 (1 H, m), 5.43 (1 H, d, J = 13.5 Hz), 4.74 (1 H, d, J = 13.5 Hz), 2.96 (1 H, m), 1.23 (3 H, d, J = 8.5 Hz), 1.02 (3 H, d, J = 8.0 Hz). 13 C NMR (125 MHz, CD3CN, 22 °C): δ 209.1, 197.5, 167.5, 165.5, 138.7, 136.2, 133.4, 132.5, 129.9, 129.8, 129.7, 128.8, 125.5, 120.9, 116.6, 64.2, 53.6, 40.7, 19.3, 18.2. ESI-MS: 440.2 [*M* + Na]+.

### Refinement

All H atoms were placed in calculated positions and refined as riding, with C—H = 0.95–1.00 Å, and N—H = 0.88 Å, and *U*_iso_(H) = 1.2–1.5 *U*_eq_(C,*N*).

### Crystal data

**Table:**

C_26_H_24_FNO_3_	*F*(000) = 880
*M**_r_* = 417.46	*D*_x_ = 1.214 Mg m^−^^3^
Monoclinic, *P*2_1_/*n*	Mo *K*α radiation, λ = 0.71073 Å
Hall symbol: -P 2yn	Cell parameters from 2549 reflections
*a* = 14.1694 (14) Å	θ = 2.4–21.8°
*b* = 9.8307 (9) Å	µ = 0.08 mm^−^^1^
*c* = 16.6367 (16) Å	*T* = 173 K
β = 99.651 (2)°	Prism, colourless
*V* = 2284.6 (4) Å^3^	0.15 × 0.10 × 0.05 mm
*Z* = 4	

### Data collection

**Table:**

Bruker SMART CCD area-detector diffractometer	3792 independent reflections
Radiation source: fine-focus sealed tube	2493 reflections with *I* > 2σ(*I*)
graphite	*R*_int_ = 0.027
φ and ω scans	θ_max_ = 24.5°, θ_min_ = 1.8°
Absorption correction: multi-scan (*SADABS*; Bruker, 2005)	*h* = −16→15
*T*_min_ = 0.990, *T*_max_ = 0.996	*k* = −11→11
10555 measured reflections	*l* = −14→19

### Refinement

**Table:**

Refinement on *F*^2^	Primary atom site location: structure-invariant direct methods
Least-squares matrix: full	Secondary atom site location: difference Fourier map
*R*[*F*^2^ > 2σ(*F*^2^)] = 0.067	Hydrogen site location: inferred from neighbouring sites
*wR*(*F*^2^) = 0.227	H-atom parameters constrained
*S* = 1.06	*w* = 1/[σ^2^(*F*_o_^2^) + (0.1129*P*)^2^ + 1.1017*P*] where *P* = (*F*_o_^2^ + 2*F*_c_^2^)/3
3792 reflections	(Δ/σ)_max_ < 0.001
275 parameters	Δρ_max_ = 0.78 e Å^−^^3^
1 restraint	Δρ_min_ = −0.68 e Å^−^^3^

### Special details

**Table:**

Geometry. All e.s.d.'s (except the e.s.d. in the dihedral angle between two l.s. planes) are estimated using the full covariance matrix. The cell e.s.d.'s are taken into account individually in the estimation of e.s.d.'s in distances, angles and torsion angles; correlations between e.s.d.'s in cell parameters are only used when they are defined by crystal symmetry. An approximate (isotropic) treatment of cell e.s.d.'s is used for estimating e.s.d.'s involving l.s. planes.
Refinement. Refinement of *F*^2^ against ALL reflections. The weighted *R*-factor *wR* and goodness of fit *S* are based on *F*^2^, conventional *R*-factors *R* are based on *F*, with *F* set to zero for negative *F*^2^. The threshold expression of *F*^2^ > σ(*F*^2^) is used only for calculating *R*-factors(gt) *etc*. and is not relevant to the choice of reflections for refinement. *R*-factors based on *F*^2^ are statistically about twice as large as those based on *F*, and *R*- factors based on ALL data will be even larger.

### Fractional atomic coordinates and isotropic or equivalent isotropic displacement parameters (Å^2^)

**Table:**

	*x*	*y*	*z*	*U*_iso_*/*U*_eq_	
C1	0.0713 (3)	0.2839 (5)	0.9806 (3)	0.0948 (13)	
H1A	0.0361	0.2691	1.0236	0.114*	
C2	0.0430 (3)	0.3814 (5)	0.9239 (3)	0.0996 (14)	
H2A	−0.0107	0.4368	0.9286	0.120*	
C3	0.0911 (2)	0.4006 (4)	0.8599 (2)	0.0769 (10)	
H3A	0.0698	0.4674	0.8196	0.092*	
C4	0.1710 (2)	0.3222 (3)	0.85420 (17)	0.0521 (7)	
C5	0.2012 (3)	0.2269 (3)	0.9127 (2)	0.0763 (10)	
H5A	0.2569	0.1745	0.9100	0.092*	
C6	0.1503 (4)	0.2071 (4)	0.9758 (2)	0.0958 (14)	
H6A	0.1705	0.1398	1.0159	0.115*	
C7	0.2586 (2)	0.2567 (3)	0.74381 (17)	0.0499 (7)	
C8	0.2898 (2)	0.3116 (3)	0.66692 (17)	0.0546 (7)	
H8A	0.2728	0.4103	0.6617	0.066*	
C9	0.2357 (2)	0.2362 (3)	0.59207 (18)	0.0617 (8)	
H9A	0.2563	0.1388	0.5948	0.074*	
C10	0.1280 (3)	0.2419 (4)	0.5882 (2)	0.0743 (10)	
C11	0.0794 (4)	0.3651 (5)	0.5882 (3)	0.1127 (15)	
H11A	0.1140	0.4482	0.5908	0.135*	
C12	−0.0191 (4)	0.3675 (8)	0.5845 (4)	0.142 (2)	
H12A	−0.0511	0.4524	0.5845	0.170*	
C13	−0.0706 (5)	0.2506 (10)	0.5810 (4)	0.146 (2)	
H13A	−0.1381	0.2531	0.5782	0.175*	
C14	−0.0231 (5)	0.1283 (8)	0.5815 (3)	0.131 (2)	
H14A	−0.0580	0.0456	0.5793	0.158*	
C15	0.0746 (3)	0.1249 (5)	0.5851 (2)	0.0931 (12)	
H15A	0.1059	0.0394	0.5855	0.112*	
C16	0.3973 (2)	0.2978 (3)	0.6729 (2)	0.0695 (9)	
C17	0.4603 (3)	0.3732 (5)	0.7404 (3)	0.1074 (10)	
H17A	0.4183	0.4299	0.7697	0.129*	
C18	0.5129 (5)	0.2730 (6)	0.8006 (4)	0.168 (3)	
H18A	0.5533	0.3225	0.8447	0.253*	
H18B	0.5531	0.2142	0.7728	0.253*	
H18C	0.4665	0.2173	0.8234	0.253*	
C19	0.5271 (3)	0.4666 (5)	0.7060 (3)	0.1074 (10)	
H19A	0.5669	0.5158	0.7505	0.161*	
H19B	0.4898	0.5319	0.6690	0.161*	
H19C	0.5681	0.4133	0.6759	0.161*	
C20	0.2594 (3)	0.2989 (3)	0.5144 (2)	0.0752 (10)	
C21	0.2747 (2)	0.2140 (3)	0.44349 (17)	0.0595 (8)	
C22	0.2713 (2)	0.0741 (3)	0.44226 (19)	0.0632 (8)	
H22A	0.2592	0.0261	0.4890	0.076*	
C23	0.2853 (3)	0.0031 (4)	0.3737 (2)	0.0784 (10)	
H23A	0.2820	−0.0934	0.3725	0.094*	
C24	0.3038 (3)	0.0743 (4)	0.3080 (2)	0.0821 (11)	
C25	0.3089 (3)	0.2124 (4)	0.3068 (2)	0.0882 (11)	
H25A	0.3221	0.2593	0.2600	0.106*	
C26	0.2945 (3)	0.2819 (4)	0.3749 (2)	0.0776 (10)	
H26A	0.2980	0.3784	0.3754	0.093*	
N1	0.21798 (16)	0.3485 (2)	0.78690 (14)	0.0535 (6)	
H1	0.2207	0.4340	0.7719	0.066 (9)*	
O1	0.26887 (16)	0.13615 (19)	0.76232 (13)	0.0648 (6)	
O2	0.2615 (3)	0.4226 (3)	0.50929 (17)	0.1281 (13)	
O3	0.4304 (2)	0.2291 (3)	0.62480 (19)	0.1088 (11)	
F1	0.3192 (2)	0.0052 (3)	0.24109 (15)	0.1311 (11)	

### Atomic displacement parameters (Å^2^)

**Table:**

	*U*^11^	*U*^22^	*U*^33^	*U*^12^	*U*^13^	*U*^23^
C1	0.107 (3)	0.106 (3)	0.087 (3)	−0.021 (3)	0.061 (3)	−0.016 (3)
C2	0.066 (2)	0.147 (4)	0.092 (3)	0.020 (2)	0.032 (2)	−0.018 (3)
C3	0.069 (2)	0.094 (3)	0.071 (2)	0.0213 (19)	0.0209 (17)	−0.0039 (19)
C4	0.0572 (17)	0.0468 (15)	0.0567 (17)	−0.0004 (13)	0.0220 (13)	−0.0059 (13)
C5	0.106 (3)	0.0581 (19)	0.075 (2)	0.0208 (18)	0.045 (2)	0.0083 (17)
C6	0.160 (4)	0.064 (2)	0.080 (3)	0.010 (2)	0.066 (3)	0.0072 (19)
C7	0.0563 (16)	0.0431 (16)	0.0535 (16)	−0.0041 (12)	0.0186 (13)	−0.0047 (12)
C8	0.0694 (19)	0.0447 (15)	0.0550 (17)	−0.0097 (13)	0.0256 (14)	−0.0043 (13)
C9	0.089 (2)	0.0455 (16)	0.0534 (17)	−0.0118 (15)	0.0210 (15)	−0.0043 (13)
C10	0.086 (2)	0.080 (2)	0.0550 (19)	−0.017 (2)	0.0062 (16)	0.0002 (16)
C11	0.095 (3)	0.098 (3)	0.139 (4)	0.002 (3)	0.001 (3)	0.005 (3)
C12	0.087 (4)	0.169 (6)	0.163 (6)	0.025 (4)	0.003 (3)	0.013 (4)
C13	0.090 (4)	0.215 (8)	0.125 (5)	−0.029 (5)	0.001 (3)	0.002 (5)
C14	0.107 (4)	0.173 (6)	0.113 (4)	−0.059 (4)	0.015 (3)	0.000 (4)
C15	0.103 (3)	0.101 (3)	0.077 (2)	−0.038 (2)	0.018 (2)	−0.002 (2)
C16	0.077 (2)	0.067 (2)	0.073 (2)	−0.0159 (17)	0.0366 (18)	−0.0126 (17)
C17	0.098 (2)	0.116 (2)	0.111 (2)	−0.0421 (18)	0.0240 (18)	−0.0169 (19)
C18	0.165 (6)	0.151 (6)	0.163 (6)	0.002 (4)	−0.051 (5)	0.006 (4)
C19	0.098 (2)	0.116 (2)	0.111 (2)	−0.0421 (18)	0.0240 (18)	−0.0169 (19)
C20	0.117 (3)	0.0525 (19)	0.061 (2)	−0.0195 (18)	0.0282 (19)	−0.0035 (15)
C21	0.070 (2)	0.0604 (19)	0.0477 (16)	−0.0125 (15)	0.0091 (14)	−0.0014 (14)
C22	0.071 (2)	0.061 (2)	0.0578 (18)	−0.0119 (15)	0.0107 (15)	−0.0049 (15)
C23	0.096 (3)	0.068 (2)	0.072 (2)	−0.0158 (19)	0.0166 (19)	−0.0157 (18)
C24	0.097 (3)	0.096 (3)	0.055 (2)	−0.012 (2)	0.0169 (18)	−0.025 (2)
C25	0.121 (3)	0.095 (3)	0.051 (2)	−0.015 (2)	0.0210 (19)	−0.0007 (19)
C26	0.112 (3)	0.068 (2)	0.0532 (19)	−0.0108 (19)	0.0178 (18)	0.0008 (16)
N1	0.0659 (15)	0.0405 (13)	0.0598 (14)	0.0020 (11)	0.0268 (12)	0.0001 (11)
O1	0.0940 (16)	0.0417 (12)	0.0663 (13)	0.0069 (10)	0.0355 (11)	0.0004 (9)
O2	0.261 (4)	0.0547 (16)	0.0844 (19)	−0.0303 (19)	0.076 (2)	−0.0051 (13)
O3	0.0924 (19)	0.129 (2)	0.121 (2)	−0.0246 (17)	0.0629 (18)	−0.0555 (19)
F1	0.195 (3)	0.128 (2)	0.0799 (16)	−0.0225 (19)	0.0503 (17)	−0.0418 (15)

### Geometric parameters (Å, °)

**Table:**

C1—C2	1.357 (6)	C14—C15	1.376 (7)
C1—C6	1.363 (6)	C14—H14A	0.9500
C1—H1A	0.9500	C15—H15A	0.9500
C2—C3	1.370 (5)	C16—O3	1.201 (4)
C2—H2A	0.9500	C16—C17	1.507 (6)
C3—C4	1.387 (4)	C17—C19	1.499 (6)
C3—H3A	0.9500	C17—C18	1.509 (7)
C4—C5	1.366 (4)	C17—H17A	1.0000
C4—N1	1.419 (3)	C18—H18A	0.9800
C5—C6	1.383 (5)	C18—H18B	0.9800
C5—H5A	0.9500	C18—H18C	0.9800
C6—H6A	0.9500	C19—H19A	0.9800
C7—O1	1.227 (3)	C19—H19B	0.9800
C7—N1	1.341 (3)	C19—H19C	0.9800
C7—C8	1.521 (4)	C20—O2	1.220 (4)
C8—C16	1.516 (5)	C20—C21	1.490 (4)
C8—C9	1.539 (4)	C21—C22	1.375 (4)
C8—H8A	1.0000	C21—C26	1.390 (4)
C9—C10	1.517 (5)	C22—C23	1.379 (4)
C9—C20	1.520 (4)	C22—H22A	0.9500
C9—H9A	1.0000	C23—C24	1.361 (5)
C10—C15	1.372 (5)	C23—H23A	0.9500
C10—C11	1.394 (6)	C24—F1	1.352 (4)
C11—C12	1.387 (7)	C24—C25	1.360 (6)
C11—H11A	0.9500	C25—C26	1.368 (5)
C12—C13	1.357 (8)	C25—H25A	0.9500
C12—H12A	0.9500	C26—H26A	0.9500
C13—C14	1.377 (8)	N1—H1	0.8800
C13—H13A	0.9500		
			
C2—C1—C6	120.0 (3)	C10—C15—C14	121.7 (5)
C2—C1—H1A	120.0	C10—C15—H15A	119.2
C6—C1—H1A	120.0	C14—C15—H15A	119.2
C1—C2—C3	120.7 (4)	O3—C16—C17	121.6 (4)
C1—C2—H2A	119.7	O3—C16—C8	120.4 (3)
C3—C2—H2A	119.7	C17—C16—C8	118.0 (3)
C2—C3—C4	119.7 (4)	C19—C17—C16	110.4 (4)
C2—C3—H3A	120.2	C19—C17—C18	112.4 (5)
C4—C3—H3A	120.2	C16—C17—C18	109.8 (4)
C5—C4—C3	119.6 (3)	C19—C17—H17A	108.0
C5—C4—N1	123.7 (3)	C16—C17—H17A	108.0
C3—C4—N1	116.7 (3)	C18—C17—H17A	108.0
C4—C5—C6	119.8 (3)	C17—C18—H18A	109.5
C4—C5—H5A	120.1	C17—C18—H18B	109.5
C6—C5—H5A	120.1	H18A—C18—H18B	109.5
C1—C6—C5	120.3 (4)	C17—C18—H18C	109.5
C1—C6—H6A	119.9	H18A—C18—H18C	109.5
C5—C6—H6A	119.9	H18B—C18—H18C	109.5
O1—C7—N1	124.0 (2)	C17—C19—H19A	109.5
O1—C7—C8	121.0 (2)	C17—C19—H19B	109.5
N1—C7—C8	115.0 (2)	H19A—C19—H19B	109.5
C16—C8—C7	110.1 (3)	C17—C19—H19C	109.5
C16—C8—C9	111.7 (2)	H19A—C19—H19C	109.5
C7—C8—C9	109.5 (2)	H19B—C19—H19C	109.5
C16—C8—H8A	108.5	O2—C20—C21	119.7 (3)
C7—C8—H8A	108.5	O2—C20—C9	118.3 (3)
C9—C8—H8A	108.5	C21—C20—C9	121.9 (3)
C10—C9—C20	108.3 (3)	C22—C21—C26	118.6 (3)
C10—C9—C8	112.2 (3)	C22—C21—C20	124.2 (3)
C20—C9—C8	110.0 (2)	C26—C21—C20	117.2 (3)
C10—C9—H9A	108.8	C21—C22—C23	120.6 (3)
C20—C9—H9A	108.8	C21—C22—H22A	119.7
C8—C9—H9A	108.8	C23—C22—H22A	119.7
C15—C10—C11	117.3 (4)	C24—C23—C22	118.5 (3)
C15—C10—C9	121.0 (4)	C24—C23—H23A	120.7
C11—C10—C9	121.7 (3)	C22—C23—H23A	120.7
C12—C11—C10	120.5 (5)	F1—C24—C25	118.3 (3)
C12—C11—H11A	119.7	F1—C24—C23	118.8 (4)
C10—C11—H11A	119.7	C25—C24—C23	122.9 (3)
C13—C12—C11	121.2 (6)	C24—C25—C26	118.1 (3)
C13—C12—H12A	119.4	C24—C25—H25A	121.0
C11—C12—H12A	119.4	C26—C25—H25A	121.0
C12—C13—C14	118.7 (6)	C25—C26—C21	121.3 (3)
C12—C13—H13A	120.7	C25—C26—H26A	119.4
C14—C13—H13A	120.7	C21—C26—H26A	119.4
C15—C14—C13	120.6 (6)	C7—N1—C4	126.8 (2)
C15—C14—H14A	119.7	C7—N1—H1	116.6
C13—C14—H14A	119.7	C4—N1—H1	116.6

### Hydrogen-bond geometry (Å, °)

**Table:**

*D*—H···*A*	*D*—H	H···*A*	*D*···*A*	*D*—H···*A*
N1—H1···O1^i^	0.88	2.10	2.959 (2)	178 (4)
C1—H1A···O3^ii^	0.95	2.43	3.371 (5)	169
C5—H5A···O1	0.95	2.52	2.963 (4)	109
C26—H26A···O2	0.95	2.41	2.735 (4)	100

Symmetry codes: (i) −*x*+1/2, *y*+1/2, −*z*+3/2; (ii) *x*−1/2, −*y*+1/2, *z*+1/2.
